# Sérgio Ferreira and *Bothrops jararaca* at the Royal College of Surgeons, London

**DOI:** 10.3390/toxins15090522

**Published:** 2023-08-25

**Authors:** Y. S. Bakhle, B. R. Ferreira

**Affiliations:** 1 National Heart and Lung Institute, Imperial College, London SW7 2BX, UK; 2 Ribeirão Preto College of Nursing, University of São Paulo, São Paulo 14040-902, Brazil; brferrei@usp.br

In 1965, Sérgio Ferreira had completed his PhD programme under the supervision of Prof Rocha e Silva, his thesis had been accepted, and he was preparing to go to England for his first post-doctoral fellowship at the Pharmacology Department at Oxford University. This department had one of the best research reputations in the UK, with Paton adding his expertise in receptor theory to the well-established groups of Blaschko, Bulbring and Vaughan-Williams, as well as a Chemical Pharmacology team led by Raymond Ing and Edward Gill. Not long before he could take up this prestigious and very attractive offer, it became clear that he would have to find a post-doctoral position in London instead, as his wife (also a University lecturer) had secured a research post at the London School of Economics (LSE). Thus, Sérgio joined John Vane in a small and relatively unknown Department of Pharmacology, sited in the Royal College of Surgeons (RCS) in Lincoln’s Fields, very close to the LSE. This was a real disappointment as the RCS Department was, at the time, much smaller than the Oxford Department, with only two research groups and no established research reputation (Figure 1). What no one knew or suspected was that, in the next decade, the RCS Department would develop a high research reputation of its own and would also be crucially involved in two of the most significant therapeutic advances of recent years, advances that are now, almost 60 years later, still providing scientifically innovative and clinically valuable insights. Sérgio was an essential part of both of these pharmacological discoveries, and his acceptance of the “second-best” post-doctoral position at the RCS turned out to be more than the best he could have hoped for, or even dreamed of, at Oxford. This unexpected success was due to Sérgio’s enthusiasm for, and commitment to, bioassay as an essential and powerful pharmacological technique and the happy chance that John Vane was an equally committed follower of Gaddum’s approach to pharmacology. Indeed, Vane had just started [1] to develop his own version—the blood-bathed organ technique (BBOT)—of Gaddum’s superfusion bioassay [2] to study the fate of a wide range of endogenous vasoactive mediators in vivo, a study that was summarized a few years later [3].

Also, in 1965, Sérgio published the first description of a bradykinin potentiating factor (BPF), a mixture of small (5–11 amino acids long) peptides isolated from the venom of the snake *Bothrops jararaca* [4], the same venom that had been used to generate and identify bradykinin itself, by Rocha e Silva [5]. Thus, when Sérgio joined Vane’s research group at the RCS, he brought with him expertise in bradykinin’s activities and metabolism, including the actions of BPF, and the world’s only sample of BPF as a freeze-dried powder, together with his belief in the value of bioassay. Not surprisingly, Vane asked Sérgio to use the BBOT technique to analyse the pharmacokinetics of bradykinin in the circulation. But first, Sérgio had to acquire this new technique and find a bioassay tissue that would respond selectively to bradykinin when superfused with blood [6]. Next, he showed that bradykinin was extensively inactivated on a single passage through the pulmonary circulation [7], thus ensuring that very little of the bradykinin that could have been generated in venous blood would survive to enter the systemic arterial circulation. However, this was a selective process, as the closely related peptide, eledoisin, was not similarly cleared, with more than 90% surviving on a single passage through the pulmonary circulation. This inactivation of bradykinin in the pulmonary circulation was more extensive and more rapid than that observed when the peptide was incubated with the animal’s blood, ex vivo, implying that the kinases involved were in the lung tissue itself (and not in the blood) but were freely accessible to the blood-borne nonapeptide. This, in turn, suggested a location for the relevant bradykininase on the surface of the pulmonary endothelium. Another critical finding was that the BPF that Sérgio had isolated from *B. jararaca* venom blocked the pulmonary inactivation of bradykinin in vivo.

In an independent study, another post-doctoral fellow, Kevin Ng, in Vane’s group was following the fate of two other plasma peptides, angiotensin I and angiotensin II, in vivo, again using the BBOT [8,9]. Somewhat unexpectedly, Ng found that angiotensin II passed through the pulmonary circulation without change, but that its precursor peptide, angiotensin I, was extensively metabolized to angiotensin II. Angiotensin I, a decapeptide, was known to be cleaved to form the octapeptide angiotensin II by the angiotensin-converting enzyme (ACE) in blood [10], but, as observed with bradykinin, the conversion of angiotensin I to angiotensin II in blood was much less than that observed in the short time taken, in vivo, to pass from the right ventricle to the left ventricle. This finding implied that, again, the relevant enzyme, ACE, appeared in lung tissue and was easily accessed by the decapeptide substrate generated in blood. These selective patterns of peptide metabolism in the pulmonary circulation induced another member of Vane’s group to prepare a cell-free fraction of lung tissue that exhibited both ACE and bradykininase activities [11]. The bradykininase activity in this fraction was inhibited by Sérgio’s BPF, and, unexpectedly, so was the lung ACE activity.

At this time, Sérgio had returned to Brazil, having completed his post-doctoral fellowship, but he immediately recognized the significance of these results in terms of physiology and pharmacology. For physiology, because all the blood passes through the pulmonary circulation, such passage would inactivate the vasodilator peptide bradykinin and convert the inactive angiotensin I to the potent vasoconstrictor angiotensin II. This meant that the pulmonary circulation was acting like an endocrine organ, secreting a net vasoconstrictor activity. As the entire cardiac output passes through the pulmonary circulation, such changes would affect all of the arterial blood circulating to the organs of the body. The pulmonary circulation was now not only the site of gas exchange but also provided a physiological means of controlling the levels of circulating endogenous vasoactive agents [12]. And BPF provided a pharmacological tool to test the physiological importance of this new lung function.

The next step was, therefore, to separate and identify the peptides in the mixture present in BPF. Sérgio achieved this in collaboration with Lewis Greene, then working at Brookhaven in the USA, and tested each isolated peptide for inhibition of bradykinin inactivation [13] and then, in collaboration with Vane’s team, as inhibitors of lung ACE [14]. The activity profiles against the two peptide substrates were almost identical, with a nonapeptide (called BPP9) being the most potent inhibitor of both activities. There was now enough evidence for Sérgio and John Vane to take to E. R. Squibb, a relatively small pharmaceutical company in the USA, and convince their research director, A. D. Welch, that it was worthwhile to look for a synthetic, non-peptide ACE inhibitor, based on the structures of the peptides (BPP) in the BPF, as a possible therapeutic agent. Although the prevailing clinical advice did not support such a possibility [15], Welch selected a biochemist, Dave Cushman, and a peptide chemist, Miguel Ondetti, to lead the search for a synthetic BPP as an inhibitor of ACE, a search which culminated in the discovery of SQ14225 [16], finally approved by the FDA in 1981 as captopril, for the treatment of hypertension. In the next few years, ACE inhibitors would be approved for a wide range of cardiovascular conditions, including congestive heart failure, post-myocardial infarction, and to delay the progress of renal failure. The clinical success of the ACE inhibitors led directly to a revival of the search for angiotensin II antagonists, which, in 1995, provided losartan as the first of many clinically effective angiotensin receptor blockers (ARBS). At present, ACE inhibitors and ARBS are first-line treatments for hypertension and allied cardiovascular disorders [17].

Sérgio’s second visit to the RCS Department in the early 1970s was as productive as his first, although the topic of his research was now the prostaglandins (PGs) rather than the plasma peptides. At this time, the development of a synthetic BPP analogue as an ACE inhibitor was firmly in the hands of Cushman and Ondetti at Squibb, and Vane’s team at the RCS were now involved with analysing the release and actions of the PGs, as relatively new, endogenous, biologically active molecules. The PGs were already known to be derivatives of a long chain, polyunsaturated fatty acid arachidonic acid, but their basic and clinical pharmacology were largely unexplored. Building on his earlier experience with the release and clearance of PGE_2_ [18], combined with the arrival of Salvador Moncada, a young, enthusiastic but inexperienced medical graduate from Honduras, Sérgio was ideally placed to contribute significantly [19] to Vane’s demonstration of the mode of action of aspirin (and other NSAIDs) as inhibitors of the biosynthesis of PGs [20]. The two papers from Vane’s group were accompanied by one from Gustav Born’s platelet group [21], and these three papers together validated the clinical use of aspirin as an anti-platelet agent. This was another example of repurposing for aspirin; the classical triad of activities (anti-pyretic, anti-inflammatory, and analgesic) for aspirin was now a quartet, with the addition of anti-aggregatory activity in platelets. Low-dose aspirin, to prevent thrombosis, was introduced soon after and, almost 50 years later, is still the standard against which new putative anti-platelet agents are compared in clinical trials. The widespread use of low-dose aspirin has generated many patient-years of real-world data which, in addition to preventing many millions of thrombotic episodes, is beginning to show additional valuable effects of aspirin in the formulations of poly-pills for cardiovascular disease [22] and, more surprisingly, to reduce the incidence of cancer [23].

However, for Sérgio, perhaps the most significant outcome of the second and last visit to the RCS Department was his work on the role of the PGs as potentiating agents in pain, seeking to explain the long-established analgesic effects of aspirin and NSAIDs. He was among the first to show that the PGs were more effective as potentiators of known algesic agents, such as histamine or bradykinin, rather than exerting a direct algesic effect themselves [24,25].

In 1974, Vane was appointed Research Director of the Wellcome Laboratories in the UK, and most of his team from the RCS accompanied him, including Sérgio and Salvador Moncada. Sérgio continued his pharmacological analysis of analgesia in the Wellcome Laboratories and later when he returned finally to his department in Brazil. Analgesia remained one of Sérgio’s main interests during the rest of his working life in Ribeirão Preto, where he built up a very productive team and, in collaboration with pharmacologists from the UK, maintained a scientific link which lasted for many years.

Gaddum, one of the founding fathers of British pharmacology, described bioassay as the unique and defining feature of pharmacology [26]. For Sérgio, although he qualified as a physician, his real vocation turned out to be pharmacology, deploying bioassay skillfully and ingeniously and, most importantly, following the results of his bioassays, even when they led him to conclusions at variance with those commonly accepted at the time. He did, indeed, carry out “crazy experiments” and express “strange opinions”, many of which were at first discounted and disregarded but later proved to be correct. Moreover, these strange pharmacological opinions had highly significant clinical consequences.

Although Sérgio worked at the RCS for only two short periods, on each occasion, he made real contributions to pharmacology and medicine. Both contributions would, nowadays, be seen as highly successful “repurposing”, reminding us that discovering new uses for old drugs has been a characteristic of pharmacology and medicine for very many years. His first visit to the RCS Department resulted in the repurposing of a bradykinin-potentiating peptide as an inhibitor of an angiotensin-converting enzyme. This led directly to the physiological repurposing of the RAS from “a truly hormonal controlling system intimately concerned with electrolyte balance”, which also “seems unlikely that the system usually operates as a direct pressor system” [27], into a major and highly effective target for the treatment of many examples of cardiovascular dysfunction. Furthermore, as a direct result of this clinical success, the physiology, biochemistry and pharmacology of the RAS were greatly expanded, and the RAS now includes a new converting enzyme, ACE2 (which itself has been repurposed into a target for anti-viral drugs); a new endogenous mediator peptide, angiotensin 1–7; and new receptors, AT_2_, Mas, and MRGPRD [28]. Although the relevance of these new RAS components to cardiovascular function and dysfunction is still under very active evaluation, what is already known has induced a revolution in cardiovascular medicine but, this time, a revolution that, unlike many others, has saved, not consumed, many millions of lives. His second visit, together with a re-focusing on the PGs, contributed to the repurposing of aspirin as a new and clinically valuable anti-platelet agent, which is still a first-line treatment in cardiovascular medicine. There is yet another repurposing for aspirin likely to emerge as an anti-cancer agent.

These indisputable advances in biomedical science and clinical medicine are the outcomes of the work of many scientists and clinicians in many countries, providing many pathways to our present state of knowledge and indications of the future. One of those pathways started almost 60 years ago with the arrival in England of a young Brazilian pharmacologist with a strong belief in bioassay and a freeze-dried extract of *B. jararaca* venom.

## Figures and Tables

**Figure 1:**
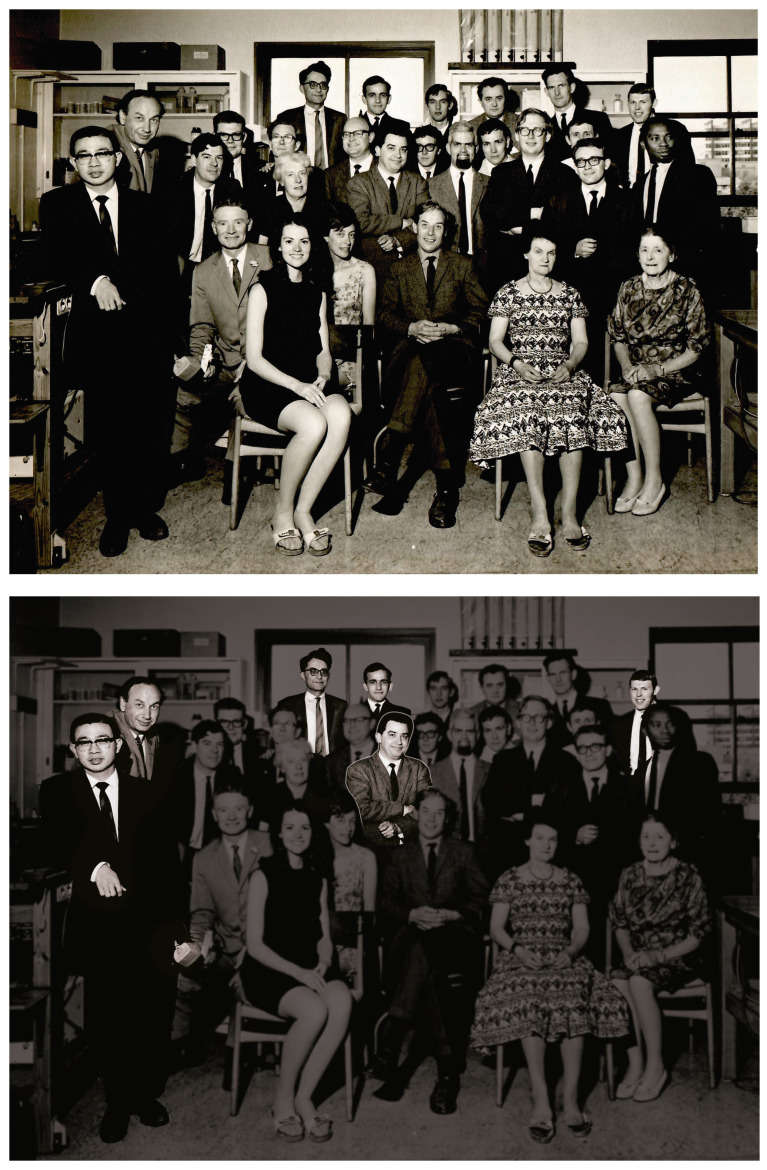
Members of the Department of Pharmacology at the Royal College of Surgeons of England, London (1965–1966) are shown in the upper photograph. In the lower photograph, the highlighting identifies Sérgio in the middle, with Kevin Ng, John Vane, Gustav Born, Rod Flower, and Bryan Smith (from left to right).

## Data Availability

Not applicable.
